# Overexpressed CISD2 has prognostic value in human gastric cancer and promotes gastric cancer cell proliferation and tumorigenesis *via* AKT signaling pathway

**DOI:** 10.18632/oncotarget.6302

**Published:** 2015-11-10

**Authors:** Lan Wang, Fei Ouyang, Xiaobo Liu, Shu Wu, Hong-mei Wu, Yuandong Xu, Bin Wang, Jinrong Zhu, Xuehu Xu, Liang Zhang

**Affiliations:** ^1^ Department of Pathogen Biology and Immunology, School of Basic Courses, Guangdong Pharmaceutical University, Guangzhou, China; ^2^ Department of Obstetrics and Gynecology, The First Affiliated Hospital, Sun Yat-sen University, Guangzhou, China; ^3^ State Key Laboratory of Oncology in South China, Guangzhou, China; ^4^ Gastrointestinal Surgery, The Third Affiliated Hospital of Guangzhou Medical University, Guangzhou, China; ^5^ Laura Biotech Co, Ltd., Guangzhou, Guangdong Province, China; ^6^ Department of Biochemistry, Zhongshan School of Medicine, Sun Yat-sen University, Guangzhou, Guangdong, China; ^7^ Center of Medical Imaging and Image-Guided Therapy, Sun Yat-sen University Cancer Center, Collaborative Innovation Center for Cancer Medicine, Guangzhou, China

**Keywords:** gastric cancer, CISD2, proliferation, tumorigenesis, AKT

## Abstract

CDGSH iron sulfur domain 2 (CISD2) is localized in the outer mitochondrial membrane and mediates mitochondrial integrity and lifespan in mammals, but its role in cancer is unknown. In the current study, we reported that CISD2 mRNA and protein expression levels were significantly upregulated in gastric cancer cells compared to normal gastric epithelial cells (*P* < 0.001). Immunohistochemical analysis of 261 paraffin-embedded archived gastric cancer tissues showed that high CISD2 expression was significantly associated with clinical stage, TNM classifications, venous invasion and lymphatic invasion. Univariate and multivariate analysis indicated that high CISD2 expression was an independent prognostic factor for poorer overall survival in the entire cohort. Overexpressing CISD2 promoted, while silencing CISD2 inhibited, the proliferation of gastric cancer cells. Furthermore, we found that silencing endogenous *CISD2* also significantly inhibited the proliferation and tumorigenicity of MGC-803 and SGC-7901 cells not only *in vitro* but also *in vivo* in NOD/SCID mice (*P* < 0.05). Furthermore, we found that CISD2 affected cell proliferation and tumorigenicity of gastric cancer cells through mediating the G1-to-S phase transition. Moreover, we demonstrated that the pro-proliferative effect of CISD2 on gastric cancer cells was associated with downregulation of cyclin-dependent kinase inhibitor p21Cip1 and p27Kip1, and activation of AKT signaling. The findings of this study indicate that CISD2 may promote proliferation and tumorigenicity, potentially representing a novel prognostic marker for overall survival in gastric cancer.

## INTRODUCTION

Gastric cancer is the most commonly diagnosed malignant cancer and is one of the most frequent causes of cancer mortality worldwide [1]. A patient has a greater chance of recovery when gastric cancer is found very early, but unfortunately it is often diagnosed in an advanced stage [2]. At later stages, gastric cancer can be only treated for symptoms but not completely cured. Unlike standard chemotherapy, novel cancer-targeting therapies are aimed at blocking the growth and spread of cancer by interfering with specific molecules (molecular targets) which are involved in the growth, progression and spread of the tumor. Currently, these cancer-targeting therapies are the focus of much anticancer drug development and the cornerstone of precision medicine. Many targeted cancer therapies have been approved by the Food and Drug Administration (FDA) to treat some types of cancer, such as Tamoxifen for breast cancer, Cetuximab for colorectal cancer and Tretinoin for leukemia. Only a few targeted therapies, such as trastuzumab and ramucirumab in the treatment of adenocarcinoma of the stomach or gastroesophageal junction, have been approved for gastric cancer. Besides only trastuzumab and ramucirumab in the treatment of adenocarcinoma of the stomach or gastroesophageal junction, few targeted therapies have been approved for gastric cancer. The development of targeting therapies requires the identification of good targets, i.e., targets that play a key role in cancer cell growth and survival. For patients with gastric cancer, additional biomarkers and therapeutic targets need to be identified to improve the early diagnosis and treatment outcomes.

CISD2 is an evolutionarily conserved gene. As one of the three members of the CDGSH iron sulfur domain protein family, CISD2 contains a transmembrane domain, a CDGSH domain and a conserved amino acid sequence for iron binding [3]. Located within a region on human chromosome 4q where a genetic component for human longevity resides, CISD2 was demonstrated primarily to be localized in the mitochondrial outer membrane and mediate mitochondrial integrity and lifespan in mammals [4-7]. Mitochondria are the cellular energy factories that generate adenosine 5-triphosphate (ATP) via oxidative phosphorylation. In wild-type C57BL/6 mice, *CISD2* mRNA expression has been shown to be decreased in older mice compared with a younger group of mice [3]. Deficiency in the CISD2 protein leads to mitochondrial degeneration, and the damaged mitochondria appear to induce autophagy in order to eliminate the dysfunctional organelles [4]. By 8 weeks, an obvious premature aging phenotype can be observed in the CISD2 knockout mice, including osteopenia, lordokyphosis, opaque eyes and blindness, lower subcutaneous fat deposition, fur de-pigmentation and skin atrophy [4]. As tumor cells have a long lifespan due to the unlimited growth capacity, all of these observations combined suggest that CISD2 plays an important role in tumor cells. CISD2 was shown to be elevated in human epithelial breast cancer cells and to reduce cell proliferation and tumor growth significantly [8]. Recently, Luxin Liu et al. reported CISD2 expression as a novel marker correlating with pelvic lymph node metastasis and prognosis in patients with early-stage cervical cancer [9]. However, the role of CISD2 in gastric cancer is still unknown [3].

In the present study, we reported that the upregulation of CISD2 mRNA and protein expression in gastric cancer cells and human gastric cancer tissues. Overexpressing CISD2 promoted, while silencing CISD2 inhibited, the proliferation and tumorigenesis of gastric cancer cells by activating the AKT signaling pathway. These findings suggest that CISD2 plays an important role in the proliferation and tumorigenicity of human gastric cancer cells and indicate that this molecule may be a valuable therapeutic target for gastric cancer.

## RESULTS

### CISD2 is upregulated in gastric cancer

**Figure 1:**
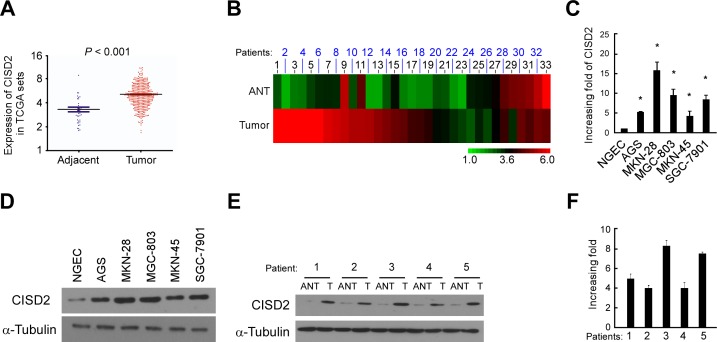
CISD2 is upregulated in gastric cancer **A.** Expression of CISD2 was frequently upregulated in 409 gastric tumor tissues (Tumor) compared with 37 adjacent normal gastric tissue samples (Adjacent) in the TCGA profile. **B.** CISD2 expression was markedly increased in 37 paired gastric tumor tissues (Tumor) and their adjacent normal tissues (Adjacent) in the TCGA profile. **C.** Real-time PCR analysis of CISD2 expression in NGEC and gastric cancer cell lines. **D.** Western blotting of CISD2 expression in NGEC and gastric cancer cell lines. **E.** CISD2 protein expression level in five paired gastric tumor tissues (T) and their adjacent normal tissues (ANT). **F.** *CISD2* mRNA expression level in five paired gastric tumor tissues (T) and their adjacent normal tissues (ANT). The average *CISD2* mRNA expression was normalized to the expression of GDPDH. α-Tubulin was detected as a loading control in the Western blot. Three independent experiments were conducted for each assay.

Through analysis of CISD2 expression in published profiles from gastric cancer patients, we found that it was upregulated in gastric cancer samples (409 cases) compared with adjacent normal tissue samples (37 cases) (*P* < 0.001, Figure 1A). To exclude the influence of differences between individuals, we further analyzed the expression of CISD2 in a total of 33 paired gastric tumor tissues in this dataset and found that it was significantly upregulated in 25 of the gastric tumor tissues compared with their adjacent normal tissues (*P* < 0.001) (Figure 1B). Real-time PCR analysis verified that CISD2 mRNA expression was indeed upregulated (by at least 4.8-fold) in cultured gastric cancer cells compared with normal gastric epithelial cells (NGEC) (Figure 1C). Furthermore, CISD2 protein expression was dramatically upregulated in all five gastric cancer cell lines tested compared to NGEC by Western blotting (Figure 1D). To examine the expression of CISD2 in clinical gastric cancer specimens, five paired gastric tumor tissues (T) and the matched adjacent non-tumor gastric tissues (ANT) were analyzed. Western blotting and real-time PCR analysis revealed that CISD2 protein and mRNA were both markedly overexpressed in the human primary gastric tumor tissues compared to the adjacent non-tumor gastric tissues (Figure 1E and 1F). Taken together, these results strongly indicate that CISD2 is upregulated in human gastric cancer.

### High CISD2 expression in gastric cancer tissues correlates with poor patient survival

**Figure 2:**
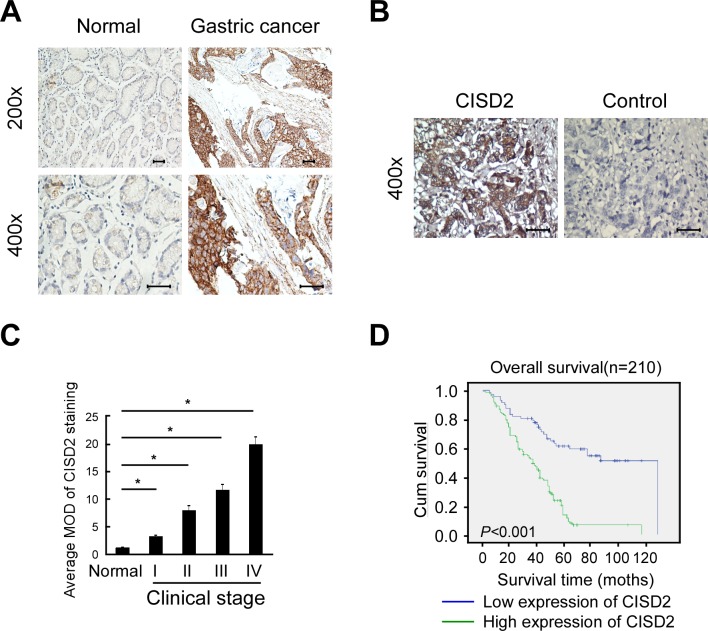
High CISD2 expression in gastric cancer tissues correlates with poor patient survival **A.** Representative images of CISD2 expression in normal gastric tissues and gastric cancer. Scales represent 50 micron. **B.** Representative images of immunostained with CISD2 (left, CISD2) or secondary antibody alone (right: control). **C.** Quantification of the average means optical density (MOD) for CISD2 in normal breast tissue and different clinical stages of breast cancer. **D.** Kaplan-Meier overall survival curves for all 210 patients with breast cancer stratified by high and low expression of CISD2.

To investigate the frequency of CISD2 upregulation in gastric cancer, we examined its expression using immunohistochemistry in 210 paraffin-embedded, archived human gastric cancer tissues, including 18 cases at clinical stage I, 50 cases at clinical stage II, 94 cases at clinical stage III and 48 cases at clinical stage IV (Table 1). These samples were stained using a CISD2 antibody and scored using a standard method (summarized in Table 2). Compared to the adjacent non-tumor gastric tissues in which CISD2 was undetectable or found to be only expressed at low levels, CISD2 was overexpressed in the gastric cancer specimens. As shown in Figure 2A, CISD2 expression was observed in the tumor cells in 188/210 (89.5%) of the samples. CISD2 was expressed at lower levels in early stage tumors (stage I-II) and at higher levels in advanced stage disease (stage III-IV). Quantitative analysis revealed that the expression of CISD2 was significantly higher in the gastric tumors samples compared to the normal gastric tissue samples (Figure 2B). Kaplan-Meier survival curves demonstrated that the overall survival of patients with high expression of CISD2 was significantly shorter than those with low CISD2 expression (Figure 2C, *P* < 0.001). Collectively, these results indicate that overexpression of CISD2 in primary gastric cancer patients correlate with poor survival.

**Table 1:** Clinicopathological characteristics of patient samples and expression of CISD2 in gastric cancer

Characteristics	Number of cases (%)
**Age(y)**	
≥60	110(52.4)
<60	100(47.6)
**Gender**	
Male	179(85.2)
Female	31(14.8)
**Clinical stage**	
I	18(8.6)
II	50(23.8)
III	94(44.7)
IV	48(22.9)
**T classification**	
T1	17(8.1)
T2	51(24.3)
T3	29(13.8)
T4	113(53.8)
**N classification**	
N0	51(24.3)
N1	63(30.0)
N2	88(41.9)
N3	8(3.8)
**Metastasis**	
No	162(77.1)
Yes	48(22.9)
**Pathologic differentiation**	
No(undifferentiated)	5(2.4)
Yes (differentiated)	205(97.6)
**Venous invasion**	
No	162(77.1)
Yes	48(22.9)
**Lymphatic invasion**	
No	54(25.7)
Yes	156(74.3)
**Vital states (at follow-up)**	
alive	62(29.5)
Dead	148(70.5)
**Expression of CISD2**	
Low expression	72(34.3)
High expression	138(65.7)

**Table 2:** Correlation between CISD2 expression and clinicopathologic characteristics of gastric cancer patients

Characteristics CISD2	CISD2		
	Low or none,no. cases	High,no. cases	*P* value
**Age(y)**			
≥60	37	73	0.885
<60	35	65	
**Gender**			
Male	64	115	0.313
Female	8	23	
**Clinical stage**			
I	11	7	0.001
II	25	25	
III	27	67	
IV	9	39	
**T classification**			
T1	11	6	0.003
T2	23	28	
T3	10	19	
T4	28	85	
**N classification**			
N0	23	28	0.017
N1	18	45	
N2	31	57	
N3	0	8	
**Metastasis**			
No	63	99	0.01
Yes	9	39	
**Pathologic differentiation**			
No(undifferentiated)	1	4	0.662
Yes (differentiated)	71	134	
**Venous invasion**			
No	63	99	0.01
Yes	9	39	
**Lymphatic invasion**			
No	25	29	0.045
Yes	47	109	

### Upregulation of CISD2 is associated with advanced clinicopathological features of gastric cancer

We analyzed the association between CISD2 and the clinicopathological features of gastric cancer. As shown in Table 2, strong associations were observed between CISD2 expression and clinical stage (*P* = 0.001), T classification (*P* = 0.003), N classification (*P* < 0.017), M classification (*P* = 0.01), venous invasion (*P* = 0.01) and lymphatic invasion (*P* = 0.045). However, the expression of CISD2 was not associated with age (*P* = 0.885), gender (*P* = 0.313) or pathologic differentiation (*P* = 0.662). Spearman analysis of correlation between CISD2 and clinicopathological features revealed that the expression of CISD2 was significantly correlated with clinical stage (*P* < 0.001), T classification (*P* < 0.001), metastasis (*P* = 0.01), venous invasion (*P* = 0.01), lymphatic invasion (*P* = 0.031) and vital status (*P* < 0.001). We also analyzed the relative risks indicated by CISD2 in the prognosis of gastric cancer. Cox-regression analysis was used to determine whether CISD2 could serve as a risk factor. As shown in Table 4, high expression of CISD2 was associated with a significantly increased risk of death in gastric cancer patients (P < 0.001) compared to those with low CISD2 expression by univariate Cox regression analyses (Table 4). Multivariate Cox regression analysis found that CISD2 could be a factor for predicting poor survival when CISD2 expression (*P* < 0.001), T classification (*P* = 0.009) and metastasis were included (*P* < 0.001) (Table 4). These results indicate a significant correlation of the expression of CISD2 with the prognosis of gastric cancer.

**Table 3:** Spearman analysis of correlation between CISD2 and clinicopathological

Variables	CISD2 expression level	
	Spearman Correlation	*p*-Value
Age	−0.014	0.836
Gender	0.074	0.284
Clinical stage	0.283	<0.001
T classification	0.249	<0.001
N classification	0.104	0.134
Metastasis	0.178	0.01
Pathologic differentiation	−0.47	0.498
Venous invasion	0.178	0.01
Lymphatic invasion	0.149	0.031
Vital states	0.434	<0.001

**Table 4:** Univariate and multivariate analyses of various prognostic parameters in patients with gastric cancer Cox-regression analysis

	Univariate analysis		Multivariate analysis		
	*p*	Regression coefficient (SE)	*p*	Relative risk	95% confidence interval
CISD2	<0.001	3.639(0.213)	<0.001	2.671	1.741-4.098
T classification	<0.001	1.853(0.092)	0.009	1.586	1.320-1.907
Metastasis	<0.001	11.608(0.225)	<0.001	7.508	4.849-11.623

### CISD2 modulates proliferation of gastric cancer cells

**Figure 3:**
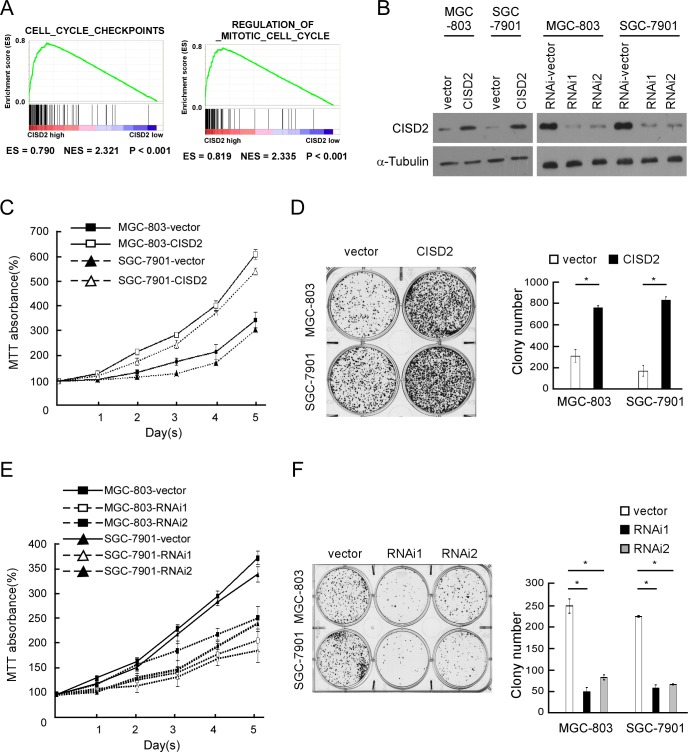
CISD2 modulates proliferation of gastric cancer cells **A.** GSEA plot showing that CISD2 expression positively correlated with cell-cycle-activated gene signatures (CELL_CYCLE_CHECKPOINTS, REGULATION_OF_MITOTIC_CELL_CYCLE). **B.** Western blot of indicated gastric cancer cells transfected with CISD2-vector, CISD2, CISD2-RNAi-vector, CISD2-RNAi1 or CISD2-RNAi2. **C.** MTT assays revealed that overexpression of CISD2 significantly increased the growth rate of indicated cells. **D.** Overexpression of CISD2 increased the mean colony number in the colony formation assay. **E.** MTT assays revealed that downregulation of endogenous CISD2 significantly reduced the growth rate. **F.** Downregulation of endogenous CISD2 reduced the mean colony number in the colony formation assay.

By determining CISD2 expression via gene set enrichment analysis (GSEA) [10, 11] the Cancer Genome Atlas (TCGA) profiles, we found that CISD2 levels were positively correlated with the proliferation by affecting genes in cell-cycle regulation. To verify results of this analysis, we ectopically overexpressed CISD2 in MGC-803 and SGC-7901 gastric cancer cells (Figure 3B). MTT assays revealed that overexpression of CISD2 significantly increased the MGC-803 and SGC-7901 cell numbers, which were approximately 2.0-fold higher at day 5 after plating compared to vector control cells (Figure 3C). Overexpression of *CISD2* also significantly increased the mean colony number in the colony formation assay (Figure 3D) compared to vector-transfected cells. Furthermore, we constructed *CISD2*-knockdown cells using two CISD2-specific shRNAs (Figure 3B). MTT and colony formation assays both revealed that knockdown of CISD2 significantly reduced the proliferation of gastric cancer cells (Figure 3E and 3F). These results indicate that CISD2 may enhance the proliferation and tumorigenicity of gastric cancer cells.

### CISD2 regulates the tumorigenesis of gastric cancer

**Figure 4:**
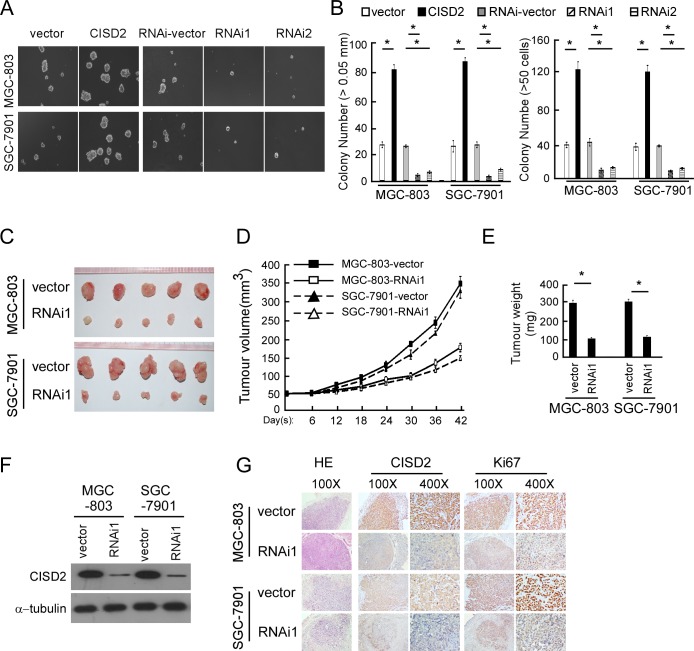
CISD2 regulates the tumorigenesis of gastric cancer (A and B) Representative micrographs **A.** and colony numbers **B.** in the anchorage-independent growth assay. Each bar represents the mean ± SEM of three independent experiments. *P < 0.05. **C.** Images of excised tumors from five NOD/SCID mice at 42 days after injection with vector-transfected cells and CISD2-RNAi1-transfected cells. **D.** Tumor volumes were measured every five days. **E.** Average weight of excised tumors. **F.** Western blotting analysis of CISD2 expression in excised xenograft tumors. **G.** Representative images of sections sliced from indicated tumors and stained with hematoxylin and eosin, anti-CISD2 and anti-Ki67 *P < 0.05.

To investigate the biological function of CISD2 in gastric cancer tumorigenesis, anchorage-independent growth ability was tested both in the CISD2-overexpressed cells and CISD2-silenced cells. As shown in Figure 4A and 4B, CISD2 could promote tumorigenesis of both MGC-803 and SGC-7901 cells, while the anchorage-independent growth ability was reduced in CISD2-silenced cells.

To validate the results of the *in vitro* tumorigenesis assays, we performed a xenograft model in BALB/C nude mice using MGC-803 and SGC-7901 cells to evaluate the effect of endogenous CISD2 *in vivo*. As shown in Figure 4C, CISD2-silenced cells had a significantly decreased ability to form tumors in nude mice compared to vector-transfected cells, as indicated by the final xenograft tumor weight, volume and the tumor growth curves (Figure 4D and 4E). Western blotting confirmed that the expression of CISD2 was significantly lower in the tumors formed by RNAi-transfected cells than those of the vector control cells (Figure 4F). The expression level of CISD2 protein and Ki67 protein were decreased in tumors formed by CISD2-RNAi1 cells (Figure 4G). Taken together, these *in vitro* and *in vivo* results demonstrate that CISD2 may play an important role in the tumorigenicity of gastric cancer cells.

### CISD2 is involved in cell-cycle G1-to-S transition

**Figure 5:**
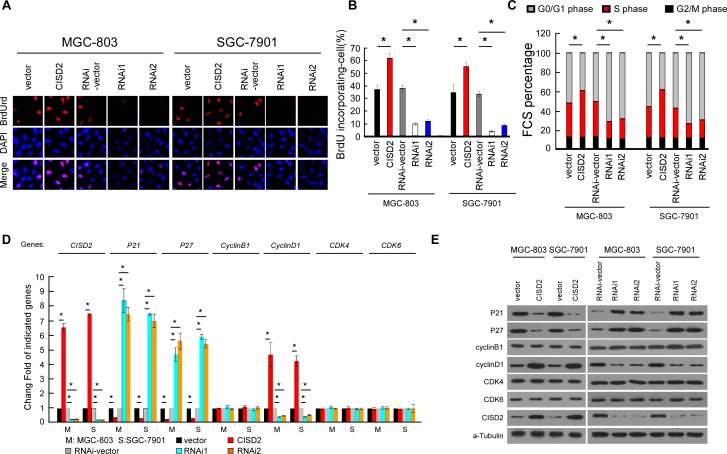
CISD2 is involved in cell-cycle G1-to-S transition **A.** and **B.** Representative micrographs **A.** and quantification **B.** of BrdUrd incorporation in indicated cells. **C.** Flow cytometric analysis of indicated cells. **D.** Real-time PCR analysis of CISD2, P21, P27, cyclinB1, cyclinD1, CDK4 and CDK6 expression in indicated cells. **E.** Western blot analysis of CISD2, P21, P27, cyclinB1, cyclinD1, CDK4 and CDK6 expression in indicated cells.

To investigate the mechanism of the proliferation-promoting function of CISD2, a bromodeoxyuridine (BrdUrd) incorporation assay was performed. As shown in Figure 5A and 5B, more than 55% of MGC-803 and SGC-7901 cells overexpressing CISD2 incorporated BrdUrd, compared with the vector-infected control cells (38% and 35% for MCG-803 and SGC-7901 control cells, respectively). Silencing CISD2 significantly decreased the proportion of cells that incorporated BrdUrd (less than 8%). Flow cytometry analysis showed that the overexpression of CISD2 significantly decreased, while silencing CISD2 increased, the percentage of cells in the G0/G1 peak but increased that in the S peak (Figure 5C), indicating that CISD2 may promote G1-to-S phase transition of gastric cancer cells. Moreover, real-time PCR and Western blotting analysis revealed that cell cycle inhibitors p21^Cip1^ and p27^Kip1^ were decreased in CISD2-transfected cells but increased in CISD2-silenced cells. Conversely, cell cycle promoter cyclinD1 was upregulated in CISD2-transfected cells. Moreover, altering expression of CISD2 had no effect on the expression of cyclinB1, CDK4 and CDK6 (Figure 5D and 5E), all of which are cell cycle promoters.

### AKT/FOXO signaling is regulated by CISD2

**Figure 6:**
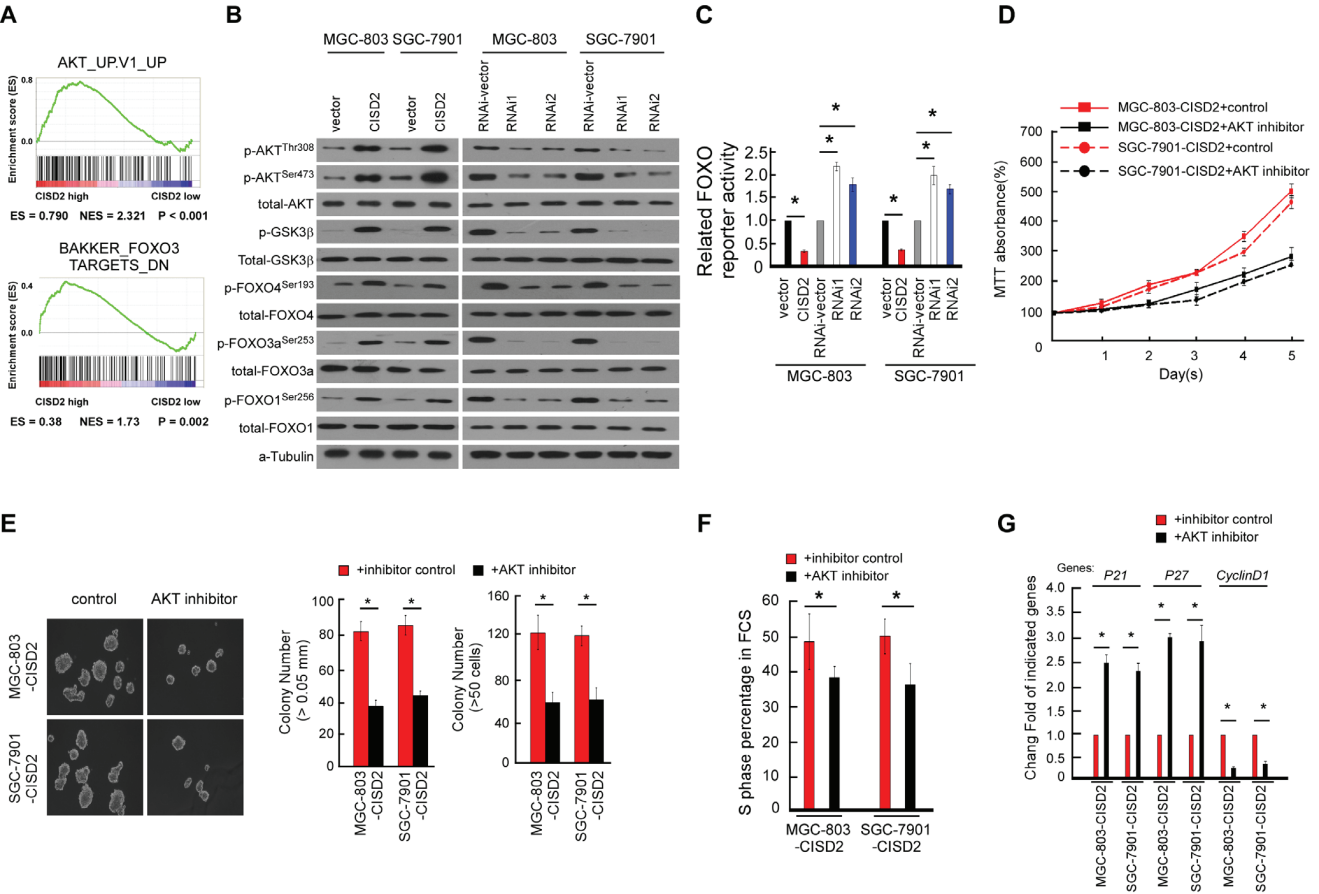
Regulation of AKT/FOXO signaling by CISD2 **A.** GSEA plot showing that CISD2 expression positively correlated with AKT-activated gene signatures (AKT_UP.V1_UP) and the FOXO3-targeted gene signatures (BAKKER_FOXO3_TARGETS_DN). **B.** Western blotting analysis of phosphorylated Akt (p-Akt), total Akt, phosphorylated GSK-3β (p-GSK-3β), total GSK-3β, phosphorylated FOXO1 (p-FOXO1-Ser256), total FOXO1, phosphorylated FOXO3a (p-FOXO3a-Ser253), FOXO3a, FOXO4 (p-FOXO4-Ser193) and total FOXO4 protein in the indicated gastric cancer cell lines. **C.** Relative FOXO reporter activity in the indicated cells. Error bars represent mean ± SD from three independent experiments. **D.** MTT assays revealed the role of AKT in the proliferation of CISD2-transduced cells. **E.** Soft agar assays revealed the role of AKT in the tumorigenesis of CISD2-transduced cells. **F.** Flow cytometric assays revealed the role of AKT in the G1-to-S transition of CISD2-transduced cells. **G.** Real-time PCR assays revealed the role of AKT in the downstream cell-cycle-associated genes of CISD2-transduced cells.

In an attempt to determine which pathway may be involved in CISD2-mediated gastric cancer progression, GSEA in the published TCGA gastric cancer database (*n* = 409) was performed. As shown in Figure 6A, we found that the CISD2 level was positively correlated with the AKT-activated gene signatures, suggesting that the AKT/FOXO3 pathway may be involved in the function of CISD2 (Figure 6A). The phosphorylation of AKT, FOXO4, FOXO3a and FOXO1 increased in CISD2-overexpressed cells but decreased in CISD2-silenced cells. In parallel, the activation of GSK3β, a common downstream target of AKT, was consistent with the phosphorylation level of AKT, suggesting that the change of AKT activity is modulated by CISD2 (Figure 6A). As shown in Figure 6B, the luciferase reporter assay demonstrated that the transcriptional activity of FOXO indeed was reduced in the CISD2-upregulated cells and increased in the CISD2-downregulated cells. Taken together, these results suggest that the observed regulation of cell cycle inhibitors p21Cip1 and p27Kip1 caused by CISD2 is associated with the AKT kinase activity and subsequently modulates the transactivation activities of FOXO factors.

To further delineate the key role of AKT in the proliferation and tumorgenesis regulated by CISD2, the kinase activity of AKT was suppressed by an AKT inhibitor (Perofosine) in CISD2-overexpressing gastric cancer cells. As shown in Figure 6D and 6E, results of the MTT assay and anchorage-independent growth assay demonstrated that the AKT inhibition decreased the cell proliferation and tumorigenesis. Consistently, the percentage of AKT inhibitor treated cells in the S phase of the cell cycle was decreased, and inhibition of AKT kinase activity abrogated the effects of CISD2 on p21, p27 and cyclinD1 (Figure 6F and 6G). Taken together, these findings suggest that the observed regulation of cell cycle and tumorigenesis caused by overexpressing or silencing CISD2 is associated with the AKT/FOXO pathway in gastric cancer cells.

## DISCUSSION

In our present study, we demonstrated that CISD2 was upregulated in gastric cancer cells and tissues. Immunostaining analysis showed that the expression level of CISD2 protein in histological sections was significantly correlated with clinical characteristics and reduced survival time of gastric cancer patients. Multivariate analysis revealed that CISD2 expression might be an independent prognostic indicator of survival in gastric cancer patients. Taken as a whole, our study suggests that CISD2 is a novel marker for the prognosis of early-stage gastric cancer. Furthermore, ectopic overexpression of CISD2 promoted, while silencing expression of CISD2 inhibited, the proliferation and tumorigenesis in gastric cancer. In addition, we found that CISD2 is involved in the G1-to-S transition of the cell cycle by regulating the AKT/FOXO signaling pathway.

Mitochondria, which serve as a central hub for responses to cellular stress as well as injury, mediate many important cellular processes, including proliferation, apoptosis, invasion and metastasis [12-14]. Despite their variability, almost all cancer cells demonstrate enhanced uptake and utilization of glucose, a phenomenon known as the Warburg effect, whereas mitochondrial activity in tumor cells is suppressed [15]. Thus, mitochondria have emerged as an important target for anticancer chemotherapy [15, 16]. As CISD2 is primarily localized to the outer mitochondrial membrane and required for maintenance of mitochondrial integrity, its deficiency can cause mitochondrial dysfunction accompanied by cell death with autophagic features [6, 17]. Moreover, CISD2 was found to be upregulated in breast cancer and early-stage cervical cancer, suggesting that it has an important role in cancers. Specifically in gastric cancer, we found here that CISD2 was upregulated in gastric tumor samples and correlated with the clinical characteristics and prognosis of the disease. Moreover, we found that ectopic expression of CISD2 promoted gastric cancer cell proliferation and tumorigenesis, while knockdown of endogenous CISD2 inhibited proliferation and tumorigenesis of gastric cancer cells. These findings indicate an important role of the mitochondria-related CISD2 protein in the development of tumors including gastric cancer.

In human cancers, AKT, a significant mediator of the cell cycle, is usually highly activated by its phosphorylation at both the Thr308 and Ser473 sites, which promotes cancer cell proliferation and migration as well as provides resistance against apoptosis [18, 19]. AKT phosphorylation inhibitors have been tested in phase I and II clinical trials and are considered a promising approach for cancer treatment [20]. More importantly, p-AKT is a crucial modulator of glucose metabolism in different cells [21, 22]. AKT was reported to be rapidly accumulated in mitochondria following phosphatidylinositol 3-kinase activation [23]. The anticancer/antiviral agent AKT inhibitor-IV has been shown to massively accumulate in mitochondria and potently disrupt cellular bioenergetics, indicating a correlation between AKT and mitochondrial activity [24]. Several prior studies have demonstrated the effect of AKT on the functions of mitochondria. The functions of hexokinase 2 (HK2), a key mediator of aerobic glycolysis and promoter of tumor growth (human glioblastoma multiforme), are dependent on AKT activation. Upon the activation of AKT, HK2 undergoes translocation to the outer membrane of mitochondria, leading to a metabolism shift from OXPHOS to glycolysis, thus promoting cancer cell survival under metabolic stress [25]. Another study found that AKT phosphorylates cytoplamic orphan receptor TR3 through its physical interaction with the N-terminus of TR3 and then blocks the translocation of TR3 from the nucleus to the mitochondria, which can elicit a pro-apoptotic effect in cancer cells [26]. Further dissection of the upstream signals revealed the contribution of the AKT/FOXO pathway in the regulation of cell cycle progression by CISD2. In the present study, CISD2 was found to activate AKT by phosphorylation at both the Thr308 and Ser473 sites. The phosphorylation and subsequent transactivation activity of FOXO factors (FOXO1 and FOXO3a), which are known activators for p27Kip1 and p21Cip1 transcription, positively changed with the expression level of CISD2. Inhibition of AKT kinase activity abrogated the effects of CISD2 on proliferation and tumorigenesis. Moreover, the phosphorylation of GSK3b was also positively regulated by CISD2. Thus, our study suggests a potential utility in CISD2-targeting strategies to deliver an anti-proliferative therapeutic effect through deactivating AKT/GSK-3β signaling and activating the FOXO/p21/p27 pathway.

## MATERIALS AND METHODS

### Cell lines and real-time PCR

NGEC and gastric cancer cell lines (AGS, MKN-28, MGC-803, MKN-45 and SGC-7901) were cultured as previously described [27]. Total RNA was extracted from cultured cells using TRIzol reagent (Invitrogen, Carlsbad, CA, USA) following the manufacturer's instructions, reverse transcribed and subjected to real-time PCR as previously described.[27] Expression data were normalized to the geometric mean of the housekeeping gene *GAPDH* to control the variability in expression levels and calculated as 2-[(CT of indicated genes) - (CT of GAPDH)], where CT represents the threshold cycle for each transcript.

### The primers used were

CISD2, forward: 5′-GCAAGGTAGCCAAGAAGTGC-3′; CISD2 reverse: 5′-CCCAGTCCCT GAAAGCATTA-3′; MYC forward: 5′-CACCGAGTCGTAGTCGAGGT-3′; MYC reverse: 5′-TTTCGGGTAGTGGAAAACCA-3′; P21 forward: 5′-GTCCACTGGGCCGAAGAG-3′; P21 reverse: 5′-TGCGTTCACAGGTGTTTCTG-3′; P27 forward: 5′-TTCATCAAGCAGT GATGTATCTGA-3′; P27 reverse: 5′-AAGAAGCCTGGCCTCAGAAG-3′; cyclinD1 forward: 5′-AACTACCTGGACCGCTTCCT-3′; cyclinD1 reverse: 5′-CCACTTGAGCTTG TTCACCA-3′; cyclinB1 forward: 5′-CAGATGTTTCCATTGGGCTT-3′; cyclinB1 reverse: 5′-GAACCTGAGCCAGAACCTGA-3′; CDK4 forward: 5′-GTCGGCTTCAGAGTTTCC AC-3′; CDK4 reverse: 5′-TGCAGTCCACATATGCAACA-3′; CDK6 forward: 5′-TGTCTG TTCGTGACACTGTGC-3′; CDK6 reverse: 5′-ATGCCGCTCTCCACCAT-3′; GAPDH forward: 5′-AC CACAGTCCATGCCATCAC-3′; GAPDH reverse: 5′-TCCACCACCCTG TTGCTGTA-3′.

### Vectors and retroviral infection

The CISD2 expression construct was generated by subcloning PCR-amplified full-length human *CISD2* cDNA into the pMSCV-retro-puro vector (Clontech, Palo Alto, CA) using the forward primer: 5′-GAAGGATCCGCCATGGTGCTGGAGAGCGTGG-3′ and reverse primer: 5′-GCCGAATTCTTATACTTCTTTCTTCTTCAG-3′. For downregulation of CISD2, two human siRNA sequences (RNAi1, CCTGAAAGCATTACCGGGTTCGCTA; RNAi2, CAGGAGATAATGTGGGTCCACTAAT) synthesized by Invitrogen (Carlsbad, CA) were cloned into the pSUPER.retro.puro plasmid (Oligoengine, Seattle, WA) to generate Psuper.retro.CISD2-RNAi. Retroviral production and infection were performed as described previously [27]. Stable cell lines expressing CISD2 or those with CISD2 silenced were selected using puromycin. The reporter plasmid for quantitatively detecting the transcriptional activity of FOXO was generated using the pGL3-Enhancer plasmid (Promega, Madison, WI, USA) as described previously [28]. In some experiments, perifosine (KRX-0401) (Cell Signaling, Beverly, MA, USA) dissolved in dimethyl sulfoxide (DMSO), was used to treat cells at indicated final concentrations (30 μM) and times.

### Western blotting

Western blotting was performed according to standard methods as previously described [29] using antibodies against CISD2 (1:1000, Proteintech, Chicago, 13318-1-AP), p-AKT, AKT, c-Myc, cyclinD1, cyclinB1, CDK4, CDK6, p21, P-FOXO1, FOXO1, p-FOXO3a, FOXO3a, P-FOXO4, FOXO4 and p27. α-Tubulin (Sigma, Saint Louis, MO, USA) was detected as a loading control.

### Immunohistochemistry

A total of 210 paraffin-embedded gastric cancer samples from patients who were histopathologically and clinically diagnosed at Sun Yat-sen University Cancer Center between 1995 and 2013 were used in the current study. Clinicopathological classification and staging were determined according to criteria of the American Joint Committee on Cancer (AJCC) [30]. Prior patient consent and approval from the Institutional Research Ethics Committee were obtained for the use of these clinical materials for research purposes. The clinicopathological features of the patients are summarized in Table 1. Normal gastric tissues were obtained and kept in our laboratory [27]. The immunohistochemistry procedure and scoring of CISD2 expression were performed as previously described [31]. Briefly, Sections adhered to slides were deparaffinized with xylene and rehydrated, submerged into EDTA antigenic retrieval buffer, treated with 3% hydrogen peroxide, incubated with 1% bovine serum albumin and then incubated with anti-CISD2 (1:100, Proteintech, Chicago, 13318-1-AP) overnight at 4°C. Normal goat serum was used as a negative control. After washed with PBST (PBS+1% tween), incubated secondary antibody (Zymed Laboratories), incubated with streptavidin-horseradish peroxidase complex (Zymed). Tissue sections were then immersed in 3.3′-diaminobenzidine and counterstained with 10% Mayer's hematoxylin, dehydrated, and mounted.

### MTT assay

Cells were seeded in 96-well plates in triplicate at the initial density of 0.2 × 10^4^ cells/well. At various time points, groups of cells were incubated with 100 μl of 0.5 mg/ml sterile MTT [3-(4, 5-dimethyl-2-thiazolyl)-2, 5-diphenyl-2H-tetrazolium bromide; Sigma] for 4 h at 37°C, after which the culture medium was removed and 150 μl of DMSO (Sigma, St. Louis, MO, USA) was added. The absorbance values were measured at 570 nm using 655 nm as the reference wavelength.

### Anchorage-independent growth assay

Five hundred cells were trypsinized and suspended in 2 ml complete media containing 0.3% agar (Sigma). The agar-cell mixture was placed on top of a layer of solidified 1% complete medium/agar. After 7 days of culture, viable colonies that contained more than 50 cells or that were larger than 0.5 mm were counted. This experiment was performed three times independently for each cell line.

### BrdUrd labeling and immunofluorescence

Cells grown on coverslips (Fisher, Pittsburgh, PA) were incubated with BrdUrd for 1 h and stained with an anti-BrdUrd antibody (Upstate, Temecula, CA, USA) according to the manufacturer's instruction. Images were acquired under a laser scanning microscope (Axioskop 2 plus, Carl Zeiss Co. Ltd., Jena, Germany).

### Flow cytometry analysis

Harvested cells in a culture dish were fixed in 80% ice-cold ethanol in PBS after washing in ice-cold PBS. The cells were then pelleted in a cooled centrifuge and resuspended in cold PBS. Cells were incubated at 37°C for 30 min followed by the addition of bovine pancreatic RNAase (Sigma) at a final concentration of 2 mg/ml and 20 mg/ml of propidium iodide (Sigma-Aldrich) for 20 min at room temperature. Total 2×104 cells were analyzed using a BD FACSCanto and data wasanalyzed using FLOWJO Software (Tree Star, Inc, Ashland, OR)

### Colony formation assays

Cells were plated in 60-mm plates (0.5 × 10^3^ cells per plate), cultured for 7 days, fixed with 10% formaldehyde for 5 min, stained with 1% crystal violet for 30 s before counting the number of colonies.

### Microarray data processing and visualization

RNA sequencing V2 profile dataset of downloaded on Nov. 29st, 2014 from The Cancer Genome Atlas (TCGA; https://tcga-data.nci.nih.gov/tcga/tcgaCancerDetails.jsp?diseaseType=STAD&diseaseName=Stomach adenocarcinoma) which contains 409 gastric tumor tissues and 37 adjacent normal gastric tissues samples. Profile data extractions were performed based on Excel and MeV 4.9 (http://www.tm4.org/mev) and Gene Set Enrichment Analysis (GSEA) was performed using GSEA 2.2.1 (http://www.broadinstitute.org/gsea).

### Xenograft tumor model

NOD/SCID mice (4-5 weeks old, 18-20 g) were purchased from Hunan SJA Laboratory Animal Co. Ltd (Changsha, Hunan, China). The Institutional Animal Care and Use Committee of Sun Yat-sen University approved all experimental procedures. Each mouse was injected on the mammary pads with vector-transfected cells (5 × 10^6^) on the left side and with CISD2-RNAi1 cells (5 × 10^6^) on the right side. Every six days, the length (L) and width (W) of tumors were measured using calipers, and their volumes were calculated using the equation (L × W^2^)/2. On day 42, the animals were euthanized, and the tumors were excised,weighed, serial sliced and stained with hematoxylin and eosin (HE), anti-CISD2 antibody and anti-Ki67 antibody. All institutional and national guidelines for the care and use of laboratory animals were followed.

### Statistical analysis

All statistical analyses were carried out using SPSS version 13.0 (SPSS Inc., Chicago, IL, USA) [27]. The associations between CISD2 expression and the clinicopathological characteristics of the patients were analyzed using the Chi-squared test. Bivariate correlations between study variables were calculated using the Spearman's rank correlation coefficient. Survival curves were plotted using the Kaplan-Meier method and compared using the log-rank test. Survival data were evaluated using univariate and multivariate Cox regression analyses [32]. A two-tailed *P*-value of less than 0.05 was considered statistically significant in all tests.

## References

[1] Jemal A, Thomas A, Murray T, Thun M (2002). Cancer statistics, 2002. CA Cancer J Clin.

[2] Choi KS, Jun JK, Suh M, Park B, Noh DK, Song SH, Jung KW, Lee HY, Choi IJ, Park EC (2015). Effect of endoscopy screening on stage at gastric cancer diagnosis: results of the National Cancer Screening Programme in Korea. Br J Cancer.

[3] Chen YF, Wu CY, Kirby R, Kao CH, Tsai TF (2010). A role for the CISD2 gene in lifespan control and human disease. Ann N Y Acad Sci.

[4] Chen YF, Kao CH, Chen YT, Wang CH, Wu CY, Tsai CY, Liu FC, Yang CW, Wei YH, Hsu MT, Tsai SF, Tsai TF (2009). Cisd2 deficiency drives premature aging and causes mitochondria-mediated defects in mice. Genes Dev.

[5] Wiley SE, Murphy AN, Ross SA, van der Geer P, Dixon JE (2007). MitoNEET is an iron-containing outer mitochondrial membrane protein that regulates oxidative capacity. Proc Natl Acad Sci U S A.

[6] Chen YF, Kao CH, Kirby R, Tsai TF (2009). Cisd2 mediates mitochondrial integrity and life span in mammals. Autophagy.

[7] Puca AA, Daly MJ, Brewster SJ, Matise TC, Barrett J, Shea-Drinkwater M, Kang S, Joyce E, Nicoli J, Benson E, Kunkel LM, Perls T (2001). A genome-wide scan for linkage to human exceptional longevity identifies a locus on chromosome 4. Proc Natl Acad Sci U S A.

[8] Sohn YS, Tamir S, Song L, Michaeli D, Matouk I, Conlan AR, Harir Y, Holt SH, Shulaev V, Paddock ML, Hochberg A, Cabanchick IZ, Onuchic JN, Jennings PA, Nechushtai R, Mittler R (2013). NAF-1 and mitoNEET are central to human breast cancer proliferation by maintaining mitochondrial homeostasis and promoting tumor growth. Proc Natl Acad Sci U S A.

[9] Liu L, Xia M, Wang J, Zhang W, Zhang Y, He M (2014). CISD2 expression is a novel marker correlating with pelvic lymph node metastasis and prognosis in patients with early-stage cervical cancer. Med Oncol.

[10] Subramanian A, Tamayo P, Mootha VK, Mukherjee S, Ebert BL, Gillette MA, Paulovich A, Pomeroy SL, Golub TR, Lander ES, Mesirov JP (2005). Gene set enrichment analysis: a knowledge-based approach for interpreting genome-wide expression profiles. Proc Natl Acad Sci U S A.

[11] Mootha VK, Lindgren CM, Eriksson KF, Subramanian A, Sihag S, Lehar J, Puigserver P, Carlsson E, Ridderstrale M, Laurila E, Houstis N, Daly MJ, Patterson N, Mesirov JP, Golub TR, Tamayo P (2003). PGC-1alpha-responsive genes involved in oxidative phosphorylation are coordinately downregulated in human diabetes. Nat Genet.

[12] Ahn CS, Metallo CM (2015). Mitochondria as biosynthetic factories for cancer proliferation. Cancer Metab.

[13] Rahman KM, Aranha O, Glazyrin A, Chinni SR, Sarkar FH (2000). Translocation of Bax to mitochondria induces apoptotic cell death in indole-3-carbinol (I3C) treated breast cancer cells. Oncogene.

[14] Bhandary B, Marahatta A, Kim HR, Chae HJ (2012). Mitochondria in relation to cancer metastasis. J Bioenerg Biomembr.

[15] Gogvadze V, Orrenius S, Zhivotovsky B (2009). Mitochondria as targets for cancer chemotherapy. Semin Cancer Biol.

[16] Wenner CE (2012). Targeting mitochondria as a therapeutic target in cancer. J Cell Physiol.

[17] Paddock ML, Wiley SE, Axelrod HL, Cohen AE, Roy M, Abresch EC, Capraro D, Murphy AN, Nechushtai R, Dixon JE, Jennings PA (2007). MitoNEET is a uniquely folded 2Fe 2S outer mitochondrial membrane protein stabilized by pioglitazone. Proc Natl Acad Sci U S A.

[18] Tazzari PL, Cappellini A, Grafone T, Mantovani I, Ricci F, Billi AM, Ottaviani E, Conte R, Martinelli G, Martelli AM (2004). Detection of serine 473 phosphorylated Akt in acute myeloid leukaemia blasts by flow cytometry. Br J Haematol.

[19] Kuo YC, Huang KY, Yang CH, Yang YS, Lee WY, Chiang CW (2008). Regulation of phosphorylation of Thr-308 of Akt, cell proliferation, and survival by the B55alpha regulatory subunit targeting of the protein phosphatase 2A holoenzyme to Akt. J Biol Chem.

[20] Knowling M, Blackstein M, Tozer R, Bramwell V, Dancey J, Dore N, Matthews S, Eisenhauer E (2006). A phase II study of perifosine (D-21226) in patients with previously untreated metastatic or locally advanced soft tissue sarcoma: A National Cancer Institute of Canada Clinical Trials Group trial. Invest New Drugs.

[21] Liu WL, Gao M, Tzen KY, Tsai CL, Hsu FM, Cheng AL, Cheng JC (2014). Targeting Phosphatidylinositide3-Kinase/Akt pathway by BKM120 for radiosensitization in hepatocellular carcinoma. Oncotarget.

[22] Coloff JL, Mason EF, Altman BJ, Gerriets VA, Liu T, Nichols AN, Zhao Y, Wofford JA, Jacobs SR, Ilkayeva O, Garrison SP, Zambetti GP, Rathmell JC (2011). Akt requires glucose metabolism to suppress puma expression and prevent apoptosis of leukemic T cells. J Biol Chem.

[23] Bijur GN, Jope RS (2003). Rapid accumulation of Akt in mitochondria following phosphatidylinositol 3-kinase activation. J Neurochem.

[24] Meinig JM, Peterson BR (2015). Anticancer/antiviral agent Akt inhibitor-IV massively accumulates in mitochondria and potently disrupts cellular bioenergetics. ACS Chem Biol.

[25] Wolf A, Agnihotri S, Micallef J, Mukherjee J, Sabha N, Cairns R, Hawkins C, Guha A (2011). Hexokinase 2 is a key mediator of aerobic glycolysis and promotes tumor growth in human glioblastoma multiforme. J Exp Med.

[26] Chen HZ, Zhao BX, Zhao WX, Li L, Zhang B, Wu Q (2008). Akt phosphorylates the TR3 orphan receptor and blocks its targeting to the mitochondria. Carcinogenesis.

[27] Song L, Wang L, Li Y, Xiong H, Wu J, Li J, Li M (2010). Sam68 up-regulation correlates with, and its down-regulation inhibits, proliferation and tumourigenicity of breast cancer cells. J Pathol.

[28] Tang ED, Nunez G, Barr FG, Guan KL (1999). Negative regulation of the forkhead transcription factor FKHR by Akt. J Biol Chem.

[29] Li J, Zhang N, Song LB, Liao WT, Jiang LL, Gong LY, Wu J, Yuan J, Zhang HZ, Zeng MS, Li M (2008). Astrocyte elevated gene-1 is a novel prognostic marker for breast cancer progression and overall patient survival. Clin Cancer Res.

[30] Carey LA, Metzger R, Dees EC, Collichio F, Sartor CI, Ollila DW, Klauber-DeMore N, Halle J, Sawyer L, Moore DT, Graham ML (2005). American Joint Committee on Cancer tumor-node-metastasis stage after neoadjuvant chemotherapy and breast cancer outcome. J Natl Cancer Inst.

[31] Zhang Z, Li J, Zheng H, Yu C, Chen J, Liu Z, Li M, Zeng M, Zhou F, Song L (2009). Expression and cytoplasmic localization of SAM68 is a significant and independent prognostic marker for renal cell carcinoma. Cancer Epidemiol Biomarkers Prev.

[32] Takata M, Yamanaka N, Tanaka T, Yamanaka J, Maeda S, Okamoto E, Yasojima H, Uematsu K, Watanabe H, Uragari Y (2000). What patients can survive disease free after complete resection for hepatocellular carcinoma?: A multivariate analysis. Jpn J Clin Oncol.

